# First identification report for amino acid composition of red algae *Gracilaria* spp. obtained from Central Java, Indonesia

**DOI:** 10.5114/bta.2024.145254

**Published:** 2024-12-19

**Authors:** Klara Kharisma Bunga Chandra, Tri Rini Nuringtyas, Tri Joko Raharjo

**Affiliations:** 1 Department of Biotechnology, Faculty of Graduate School, Universitas Gadjah Mada, Yogyakarta, Indonesia; 2 Faculty of Biology, Universitas Gadjah Mada, Yogyakarta, Indonesia; 3 Department of Chemistry, Faculty of Mathematics and Natural Sciences, Universitas Gadjah Mada, Yogyakarta, Indonesia

**Keywords:** LC-HRMS, peptides, *Gracilaria*, red seaweed, identification

## Abstract

*Gracilaria* is a genus of red algae found mainly in Asia. Various species of *Gracilaria* are distributed throughout Indonesia’s marine waters, especially in coastal areas, and are cultivated for research and economic purposes. *Gracilaria* mainly consists of polysaccharides and pigments, which have hindered protein identification. The unique protein expressions have never been analyzed using a proteomic approach, and no reports are available on *Gracilaria* spp. amino acid sequences in Central Java. Based on this background, we aimed to explore *Gracilaria* protein characterization using unique peptide sequence analysis via LC-HRMS. The usage of liquid chromatography coupled with high-resolution mass spectrometry (LC-HRMS) has been growing in biomedical and environmental sciences, offering high accuracy in protein detection. We followed the LC-HRMS standard protocol with an optimized precipitation procedure. TCA/acetone precipitation was used for protein purification, after which the precipitate was subjected to protein digestion to obtain small peptide fractions. Protein analysis results are presented as protein concentrations, molecular models, and peptide sequences. This experiment identified four sequences derived from the Rhodophyta database: TKKILDK (845.5455 Da), TVKSLLTK (889.5717 Da), ILVKTLK (814.5761 Da), and TGcGRSKR (921.4683 Da). This study reveals peptide sequences for *Gracilaria* spp., showing similarities with other red algae species, along with the functions of the peptide sequences. Furthermore, amino acid models of secondary structures were provided to support our findings.

## Introduction

*Gracilaria* spp. is a type of macroalgae in the Rhodophyta group that grows in coastal zones across Asia. It has distinct morphological characteristics, including an erect or weakly drooping thallus. This species exhibits subdichotomous, lateral, radial, or irregular branching with flat-axis-shaped branches. *Gracilaria* attaches to solid substrates with small discoidal holdfasts or lives on sandy bottoms, with the thallus partially immersed in the sand (Nur et al., 2018). It grows abundantly in tropical regions, particularly in Indonesia, where some species are cultivated for their economic value. This species is used as a polymer material for gelling agents and hydrocolloids. According to the Indonesian seaweed roadmap data from the Ministry of Maritime Affairs and Fisheries (2018), *Gracilaria* spp. ranks as the second largest seaweed commodity after *Eucheuma* spp. For comparison, global seaweed production was around 30 million tons in 2016, with Indonesia contributing almost 40%, particularly as a producer of red seaweed. Research by Meriam et al. (2015) reported that the distribution of *Gracilaria* spp. in Indonesia includes Aru Island, Makassar, Nusakambangan, Manado, and Gorontalo, and it is also found on Teluk Awur Beach in Jepara and Krakal Beach in Yogyakarta (Pramesti et al., 2016). This species is distributed in the intertidal zones of Gunung Kidul Beach, with relatively high densities. *Gracilaria* spp. has been widely analyzed for its metabolite diversity due to its potential as a food ingredient and its active compounds, such as lectins (Naik and Kumar, 2022), secondary metabolites (Kasanah et al., 2018), and mycosporine-like amino acids (Vega et al., 2021).

Despite its economic significance, the complete protein characteristics of *Gracilaria* spp. remain undetermined. *Gracilaria* mainly consists of polysaccharides and pigments, including pigment-binding proteins, which have posed challenges for protein identification. Proteomic analyses of unique protein expressions in *Gracilaria*, especially in Central Java or Indonesia, are sparse. Protein identification studies are limited, partly due to the perception of algal proteins as waste products. However, some promising algal peptides have been discovered, such as the active phycobiliprotein peptide from *Gracilaria crassa* from Indian waters (Sudhakar et al., 2015). Moreover Conde et al., (2013) conducted exploratory research on active peptide compounds with molecular weights of 31.4, 69.5, and 92.7 kDa, which demonstrated antioxidant activity. The nutritional quality of amino acids is largely determined by their quantity, proportion, and availability. Algal protein analysis is commonly used to identify new protein sources with bioactive potential or for dietary supplementation. The amino acid composition of seaweeds has frequently been studied and compared to other foods. For most seaweeds, aspartic and glutamic acids constitute a large part of the amino acid fraction (Gressler et al., 2010). In the *Gracilaria* and *Laurencia* species, protein contents range from 5.6 to 24% and 2.7 to 24.5%, respectively (Marinho Soriano et al., 2007). Some *Gracilaria* species, such as *Gracilaria verrucosa* (known as “Ogonori”), are used as food; *G. verrucosa* is a commonly consumed red alga in Japan. In some red algae, the protein fraction can represent 2.7 to 47.0% of the plant’s dry weight (Dawczynski et al., 2007). Exploration of active compounds in *Gracilaria* in Indonesia has not yet been undertaken. Research by Kasanah et al., (2018) explored the antibacterial properties of *Gracilaria edulis*, though the focus was on secondary metabolites. Despite sporadic studies on red algae proteomics, protein expression exploration can inform screenings for active potential. In line with this, the emergence of high-throughput sequencing technologies creates opportunities for whole-peptide sequencing projects aimed at gaining novel insights into.

## Experimental section

### Procedure

#### Preparation and extraction of Gracilaria spp.

*Gracilaria* spp. was obtained from Balai Besar Perikanan Budidaya Air Payau (BBPBAP) Jepara. Freeze-dried samples were manually crushed using a mortar and pestle. Phosphate buffer (500 ml, pH 7) was added to 30 g of the crushed, dry sample at room temperature for precipitation. The sample was centrifuged at 6500 rpm for 30 min, and the cells were removed. Samples were analyzed for fat, crude protein, total carbohydrates, moisture, minerals, and ash according to the AOAC 2002 method (Paez et al., 2016). The Kjeldahl method was used to determine crude protein content, with a nitrogen conversion factor of 4.92.

#### Protein precipitation with TCA/acetone

**Fig. 1:**
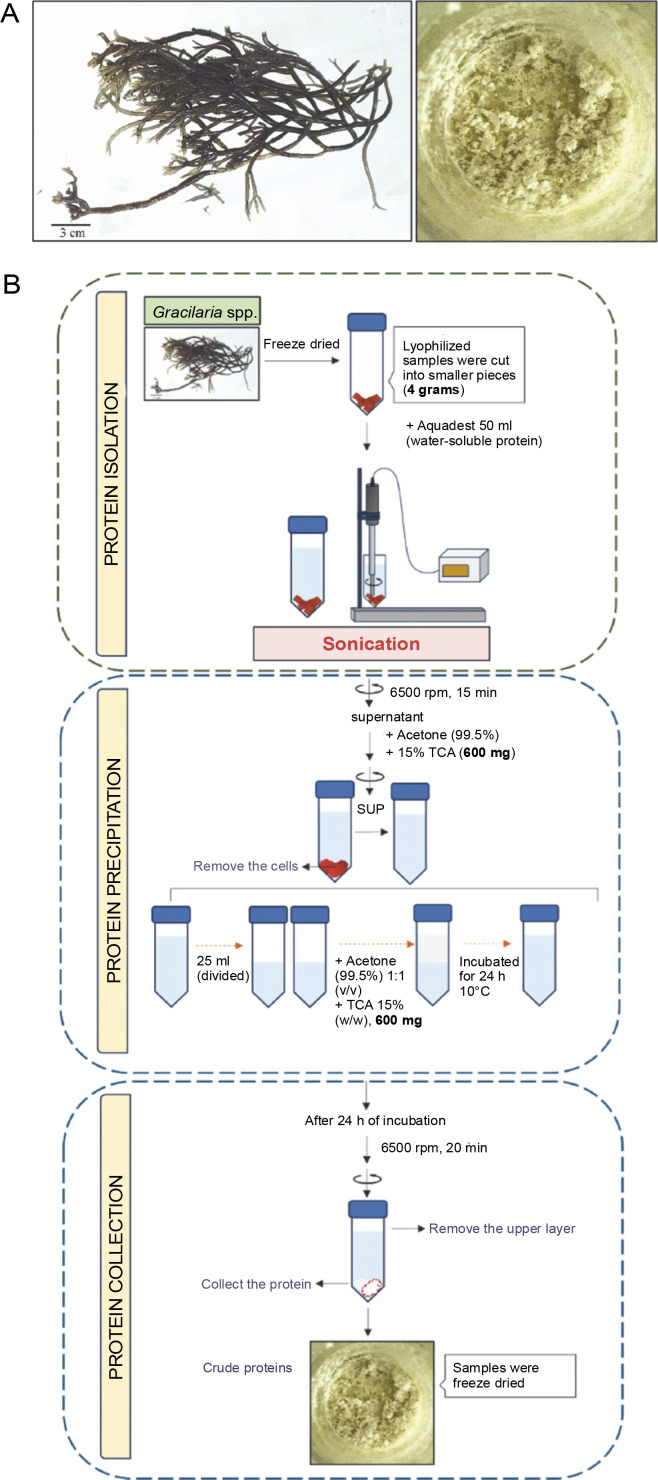
(A) Sample morphology (left) and protein extracts (right) followed by the method illustrations (B) of protein refinement by using optimized TCA/aceton methods

Protein precipitation was conducted using a TCA/acetone protocol for LC-HRMS preparation. The supernatant obtained from the extract was mixed with sterile distilled water (Oxoid). The homogenate was then placed in an Eppendorf tube, centrifuged for 5 min at 6500 rpm, and the supernatant (protein extract) was pipetted into a new tube. The study used 15% (v/v) TCA (Trichloroacetic acid)/acetone. Cold TCA/acetone was added to the protein extract, placed on ice for 5 min, centrifuged at 6500 rpm for 3 min, and the supernatant was discarded. The protein precipitate was washed twice with 80% acetone, centrifuged each time, then air-dried for 1–3 min, and dissolved in ammonium bicarbonate (NH_4_)HCO_3_ for protein analysis (Fig.1).

#### Algal protein hydrolysis

**Fig. 2:**
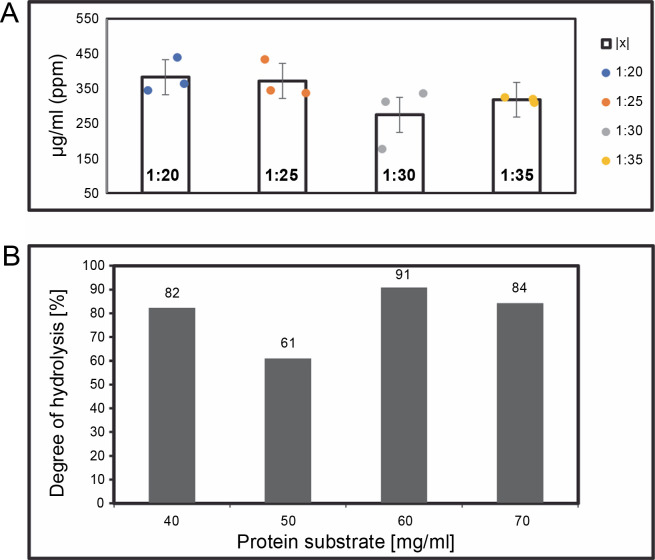
(A) The yield of > 3 kDa peptide hydrolysate concentrations (μg/ml) on various enzyme ratios, after 24 h of digestion by using Trypsin protease; (B) degree of hydrolysis was calculated following the procedures mentioned in the method section


Protein was dissolved in ammonium bicarbonate (pH 7 (NH_4_)HCO_3_). Trypsin was added at enzyme-to-substrate ratios of 1 : 20, 1 : 25, 1 : 30, and 1 : 35 (w/w) and hydrolyzed at 30°C for 24 h with constant agitation. The most efficient ratio was used for LC-HRMS characterizations (Fig. 2). The enzyme was deactivated by incubating in an oven at 90°C for 10 min. Hydrolysates were filtered using 3 kDa MWCO (Molecular Weight Cut-Off) Amicon ultrafiltration units, and centrifuged at 3,000 rpm for 1 min at 4°C; the supernatant was then collected for further analysis. Hydrolysis rates were measured as the percent of absorbance before and after enzymatic hydrolysis, calculated as:

$$\frac{\text{D}1\;(\text{peptide residue after 3MWCO filtration})}{\text{D}2\;\left(\text{the unhydrolyzed protein before filtration}\right)}\times 100\%$$

Protein concentration was determined using the Bio-Rad Protein Assay method based on Bradford’s method. To achieve a concentration of 4 mg/ml, 1 ml of distilled water and 200 μl of Bio-Rad dye (BioRad) were added. A 25 μl aliquot of this solution was transferred to a 96-well plate, and absorbance was measured at λ = 595 nm. BSA (bovine serum albumin, Bio-Rad) standards at concentrations of 10, 15, 20, 25, 30, and 35 μg/ml were used to create a standard curve. All tests were performed in triplicate.

#### LC-HRMS analysis

A total of 5 μl of hydrolysate sample (maintained at 5°C) was injected onto an Acclaim^®^ PepMap column (C18, 75 μm × 150 cm, with a particle size of 2 μm and a pore size of 100 Å). Peptides in the column were eluted using two mobile phases: mobile phase A (water with 0.05% formic acid) and mobile phase B (water: acetonitrile 20 : 80 with 0.1% trifluoroacetic acid, TFA). Mobile phases A and B were used with a specified gradient at a flow rate of 0.3 μl/min. Mass Spectrometry (MS) and MS/MS analyses were conducted using the NSI ionization method, with nitrogen as the sheath and auxiliary gases, set at 10 and 5 units, respectively. The NSI probe was set at 4000 V, and the ion transfer tube at 300°C. MS detection was performed in positive ion mode with high resolution and accurate mass settings. For MS/MS peptide analysis, the m/z range was set from 350 to 1800 using full-MS dd-MS2 mode. The full-MS parameters included a resolving power of 140,000 (FWHM), an automatic gain control target of 3 × 10^6^, and a maximum ion injection time of 100 ms. The dd-MS2 parameters were set with a resolution of 17,500 (FWHM), an automatic gain control target of 1 × 10^5^, a maximum ion injection time of 105 ms, a loop count/topN of 20, an isolation window of 1.2 m/z, and a collision energy of 27. MS analysis was performed over 90 min. Chromatograms were generated using Thermo Scientific’s Xcalibur software, and sequence identification was carried out with Thermo Proteome Discoverer 2.5. The UniProt protein database was used for analysis (https://www.uniprot.org/) – Table 1.

**Table 1:** Proximate analysis of *Gracilaria* spp. composition

Compositions	Quantity [dry weight/weight%]
Protein	10.46%
Carbohydrate	44.68%
Water	8.76%
Ash	36.10%
Lipid	0%

The experiment was conducted by triplicate (*n* = 3), and the dry weight for whole algae was 10 g

### Molecular prediction method

The 3D secondary structures of potential peptides were predicted using the PEP-FOLD v4 web server (https://bioserv.rpbs.univ-paris-diderot.fr/services/PEP-FOLD/). The primary sequence was submitted in single-letter code, and the program predicted the 3D conformation by assembling predicted conformations of short local sequences using a greedy algorithm based on a coarse-grained energy score. Protein structures were visualized using Discovery Studio 2021.

## Result and discussion

### Extraction and hydrolysis results

*Gracilaria* spp. obtained from Balai Besar Perikanan Budidaya Air Payau (BBPBAP) Jepara is a cultivated Rhodophyta species with a cylindrical shape and branching, red-brick coloration, with some parts showing green color. The sterile plant from BBPBAP cultivation showed intense branching with newly regenerated branchlets. After freeze-drying, the sample morphology changed to a pale, thick texture. Proximate analysis indicated a protein content of approximately 10.46% (w/w). The TCA/acetone method was employed for protein purification, where TCA addition forms a hydrophobic structure that is insoluble in acetone and water.

The dry weight of protein samples purified from 4 g was found to be less than 10%, or around 400 mg. Previous work on protein precipitation using PBS and TCA reported purified protein contents of *Gracilaria acerosa* at 31.07%, *Halimeda macroloba* at 28.94%, *Halimeda tuna* at 23.12%, and *Cladophora glomerata* at 20.38 ± ± 0.73% of the total dry weight (Boob et al., 2010). The addition of TCA was performed according to the method described by Niu et al. (2018). The darker color observed in the protein precipitate was predicted to indicate carbohydrate-binding proteins. Due to the unique characteristics of algae, some pigments are tightly bound to proteins, leading to the co-precipitation of other compounds. The protein content of red algae varies with factors such as season and environmental growth conditions.

The ratio described above represents enzyme (mg) to protein (mg) in terms of protein digestion. Hydrolysates were filtered using an Amicon^®^ ultrafiltration column with a 3 kDa MWCO. Peptide measurements were carried out using the BCA standard (Bichinnoic Acid). Significant differences were validated using the Steel-Dwass test, with results showing no significant difference between samples (*P*-value: 0.005).

Protein digestion ratios of 1 : 20, 1 : 25, 1 : 30, and 1 : 35 (w/w μg) were tested in triplicate samples (*n* = 3) in single applications. Analysis in R-Studio revealed no significant difference (Bartlett test; *P*-value: 0.005, Steel-Dwass; *P*-value: 0.005), so the 1 : 20 ratio was used for further analysis. This finding suggests that an increased enzyme amount correlates with a higher protein yield. The 1 : 30 enzyme digestion ratio yielded the lowest hydrolysis degree on average. However, the enzyme ratio did not directly influence digestion quality or hydrolysis degree. This study suggests that ratios between 1 : 20 and 1 : 35 achieve complete protein cleavage, and a 1 : 20 enzyme concentration is more efficient in terms of cost and yield. Trypsin protease, which has active groups of serine, histidine, and asparagine, exhibits high cleavage specificity and stability under various conditions, typically cleaving at C-terminal arginine or lysine residues (Liang et al., 2019).

### Peptide identification and characterization

**Fig. 3:**
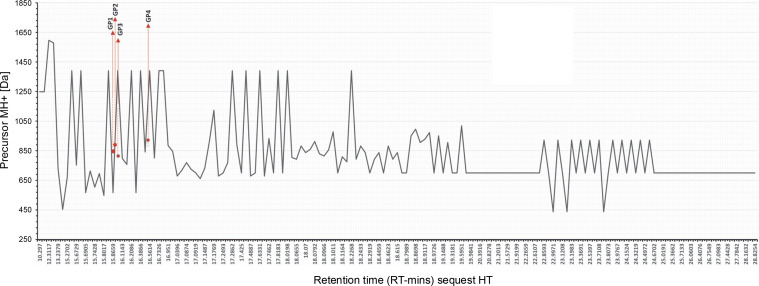
MS/MS precursor MH+ [Da] data and retention time reading request HT; the exact position of the reading sequest is marked by red line; GP1, GP2, GP3, GP4; we performed analysis by using LC-HRMS, the data was obtained from Xcalibur Thermo Fischer-Scientific^®^ with refinement graphics by using Excel; the rhodophyta database was acquired from uniport.org

An enzymatic hydrolysate rich in small peptides was obtained from a protein extract. Mass spectrometry analysis revealed spectra with four prevalent proteins identified (Table 2). The molecular size distribution profiles and physical properties of peptides from *Gracilaria* spp. are shown in Figure 3. According to the chromatogram, the hydrolysate was composed of small peptides (<1000 Da). This study focused on hydrolysate fractionation, which limited whole peptide identification. We depicted the molecular weight distribution in comparison with retention time in Figure 3, with peptides appearing at RT 17.0216 (GP1), 17.3896 (GP1), 18.0966 (GP3), and 24.5836 (GP4), respectively. Other spectra showed various MH+ scores, with many unidentified peptides when compared to the Rhodophyta database.

**Table 2:** LC-HRMS peptide sequences from *Gracilaria* spp. hydrolysate and its physical properties

ID protein	Master accesion number	XCorr value	PSMs ambiguity	Matched sequences	[MH+] Da sequences	MW [kDa] proteins	# AAs	pI	RT [min]
GP1	A0A7S0ZJV9	0.10	selected	TKKILDK	845.5455	30.4	76	9.66	17.0216
GP2	A0A1Z1M242	0.11	selected	TVKSLLTK	889.5717	8.9	106	10.5	17.3896
GP3	A0A1Z1ME08	0.74	selected	ILVKTLK	814.5761	15.8	268	6.93	18.0966
GP4	A0A7S3ENZ9	0.86	unambiguous	TGCGRSKR	921.4683	11.7	134	7.71	24.5836

* High-resolution data set; the XCorr confidence threshold or value rejection is 2.6; all data provides high confidence with the Xcorr value > 2.6

All spectra readings are shown in Table 2, displaying the number of identified peptide spectrum matches (PSMs) from all searches, including redundantly identified PSMs. LC-HRMS data shown in Table 5 confirmed one unambiguous amino acid sequence, TGcGRSKR (Thr-Gly-c(modified)-Gly-Arg-Ser-Lys-Arg), with a carbamidomethyl modification. Other PSM scores met the selected criteria for three sequences, filtered by Maximum Delta Cn (0.01) or Maximum Rank, with a rejection score of 0.05. The master accession number column offers additional details. The comparison between Da sequences and protein molecular weight (kDa) showed a rate coverage of below 10% across all sequences. Notably, peptide sequences in this study have a low molecular weight (< 3 kDa), aligning with our separation using Amicon ultrafiltration, which filtered hydrolysate with a low molecular weight under 3 kDa.

**Fig. 4:**
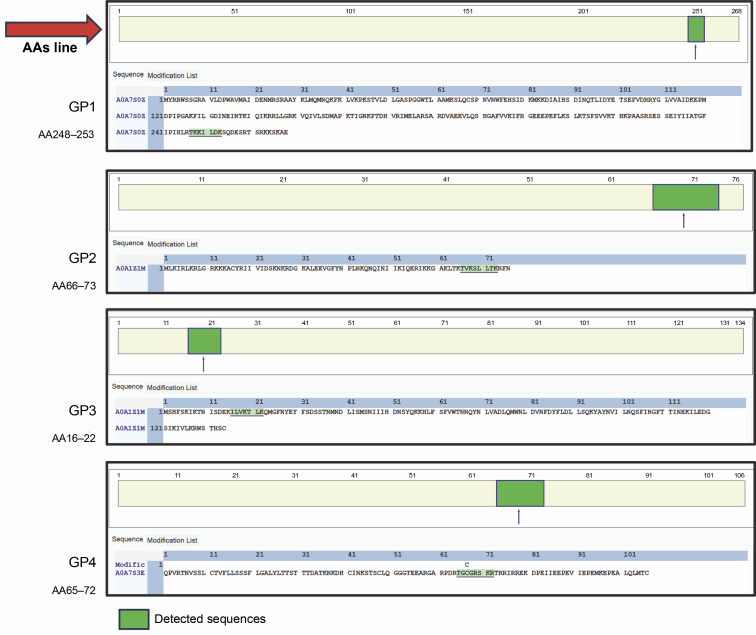
Cleavage sites of peptides reading sequences from trypsin hydrolysis

The accuracy of the data set provided in this experiment represented the cleavage sites of trypsin digestion. The data shown in Figure 3 performed arginine (R) and lysine (K) end for each site. Cutting proteins with the enzyme trypsin in the right mass range for MS provides clear peptide fragmentation spectra results. Peptides are cut with the trypsin enzyme to produce precursor ions with double charges that are easily fragmented (Steen and Mann, 2004). Figure 4 highlights coverage of identified peptides, though limited protein yield after digestion affected spectra performance. Table 3 details spectra identified as protein molecules from Rhodophyta. Sequence determination is guided by the principle of neighboring peaks. Each peptide fragment in a series distinctively appears from its cutting residue (isotope mass) of one amino acid. In principle, it is therefore possible to determine the amino-acid sequence by considering the mass difference between closer peaks in a series of spectra, with the accuracy score number 10^−4^.

**Table 3:** TKKILDK (GP1) y^+^ and b^+^ peak of LC-HRMS spectra

#1	_b_+	_b_2+	Sequence	_y_+	_y_2+	MW [Da]	#2
1	102.055	51.53112	T				7
2	230.1499	115.5786	K	744.4978	372.7525	128.095	6
3	358.2449	179.6261	K	616.4028	308.7051	128.095	5
4	471.3289	236.1681	I	488.3079	244.6576	113.0841	4
5	584.413	292.7101	L	375.2238	188.1155	113.0841	3
6	699.44	350.2236	D	262.1398	131.5735	115.027	2
7			K	147.1128	74.06004	147.1128	1

The obtained m/z values for each amino acid from spectra of the TKKILDK sequence are shown in the following Table 3. The determination of sequence was acquired by collecting reduction ion of monoisotopic mass either from b+ (forward) or y+ (reverse) which shows the directions. Tryptic peptides usually become doubly protonated and are then described – M is the mass of the peptide and H+ is the mass of a proton. The reduction score of neighbour peaks showed a mono-isotope mass score which defined the molecular weight of specific amino acids. The ions score is called b-ions if the charge is retained by the amino-terminal part of the peptide and y-ions if the charge is retained by the carboxy-terminal part. The decision of the chosen sequence was conducted by using PSM ambiguity filters. The sequence is known to be a part of rRNA methyltransferase 2 mitochondrial RNA isolated from *Timspurckia oligopyrenoides*, with the order Porphyridiales> The original sequence for single cells identified is 268 amino acids, which concludes that this section coverage is about 2.61%. Roles of this protein express catalysis of the transfer of a methyl group to an acceptor molecule and modify rRNA at certain stages of the assembly (Lopez Sanchez et al., 2020). rRNA methyltransferase is generally a well-conserved protein with close homologs in prokaryotes, archaea, and eukaryotes. Of note, this sequence TKKILDK showed significant similarity with other red algae families (Beveren, 2023).

The spectra showed In the TVKSLLTK sequence, the m/z values for each amino acid are obtained which are shown in the following Table 4. In some cases, defining leusine and isoleusine was intriguing. Complex isoforms with similar molecular weight can be confusing, however, the refining data alignment through LC-HRMS signals an autocorrelation to determine this overlap. This peptide covers 30S ribosomal protein S16 from chloroplast of about 10.53% in high similarity with *Acrosorium ciliolatum*. Despite the limited coverage, chloroplast peptide composition is considered a conserved region among other species across Rhodophyta. *Gracilaria* spp. chloroplast genome has evolved and been identified by previous studies (Hagopian et al., 2004). The chloroplast genomes across the subphylum *Eurhodophytina* are highly conserved on the genome architecture which leads to its protein expression similarity. The chloroplast genomes which are then translated into protein, hold substantial information for improving the resolution of phylogenetic relationships at all taxonomic hierarchical levels (Ng et al., 2017).

**Table 4:** TVKSLLTK (GP2) y^+^ and b^+^ peak of LC-HRMS spectra

#1	_b_+	_b_2+	Sequence	_y_+	_y_2+	MW [Da]	#2
1	102.055	51.53112	T				8
2	201.1234	101.0653	V	788.524	394.7657	99.06842	7
3	329.2183	165.1128	K	689.4556	345.2314	128.095	6
4	416.2504	208.6288	S	561.3606	281.184	87.03203	5
5	529.3344	265.1709	L	474.3286	237.6679	113.0841	4
6	642.4185	321.7129	L	361.2446	181.1259	113.0841	3
7	743.4662	372.2367	T	248.1605	124.5839	101.0477	2
8			K	147.1128	74.06004	147.1128	1

The peptide fragment ILVKTLK read ile-leu-val-lys-thr-leu-lys showed the distinguished encryption between isoleusine and leusine as shown in Table 5. These peptides are encoded inside chloroplast namely YCF35 protein isolated from *Chondrus crispus*, however, the function of this protein is not yet classified. Therefore, this sequence model is grouped as an uncharacterized protein.

**Table 5:** ILVKTLK (GP3) y^+^ and b^+^ peak of LC-HRMS spectra

#1	_b_+	_b_2+	Sequence	_y_+	_y_2+	MW [Da]	#2
1	114.0913	57.54931	I				7
2	227.1754	114.0913	L	701.492	351.2496	113.0841	6
3	326.2438	163.6256	V	588.4079	294.7076	99.06841	5
4	454.3388	227.673	K	489.3395	245.1734	128.095	4
5	555.3865	278.1969	T	361.2446	181.1259	101.0477	3
6	668.4705	334.7389	L	260.1969	130.6021	113.0841	2
7			K	147.1128	74.06004	147.1128	1

The other interesting sequence is TGcGRSKR, which showed the highest molecular weight among others, obtained from *Rhodosporus marinus*. Accordingly, the protein is grouped as an uncharacterized protein isolated from the membrane. Table 6 showed a unique post-translational modification C-Carbamidomethyl of y6+ (763.3992) and the mass of y5+ (603.3685) with the mass value of 160.0307 Da for carbamidomethyl (c). This post modification is recognized by the algorithm as a consequence of *satellite ions* or the fragments ion following the neighbor peak (Carrasco-Castilla et al., 2012; Charlesworth et al., 2013; Hussein et al., 2020; Pergande and Cologna, 2017). Cysteine Carbamidomethylation (Cysteine CAM) is a modification due to a reaction with iodoacetamide and is used to block cysteine from oxidation (Murphy et al., 2021; Patwary et al., 2023). This sequence also confirmed enzyme end-point cuts in arginine (R).

**Table 6:** TGcGRSKR (GP4) y^+^ and b^+^ peak of LC-HRMS spectra

#1	_b_+	_b_2+	Sequence	_y_+	_y_2+	MW [Da]	#2
1	102.055	51.53112	T				8
2	159.0764	80.04185	G	820.4206	410.714	57.02146	7
3	319.1071	160.0572	C–Carbamidomethyl	763.3992	382.2032	160.0307	6
4	376.1285	188.5679	G	603.3685	302.1879	57.02147	5
5	532.2296	266.6185	R	546.3471	273.6772	156.1011	4
6	619.2617	310.1345	S	390.2459	195.6266	87.03202	3
7	747.3566	374.182	K	303.2139	152.1106	128.095	2
8			R	175.119	88.06311	175.119	1

### Prediction model of Gracilaria spp. peptides compartment

**Fig. 5:**
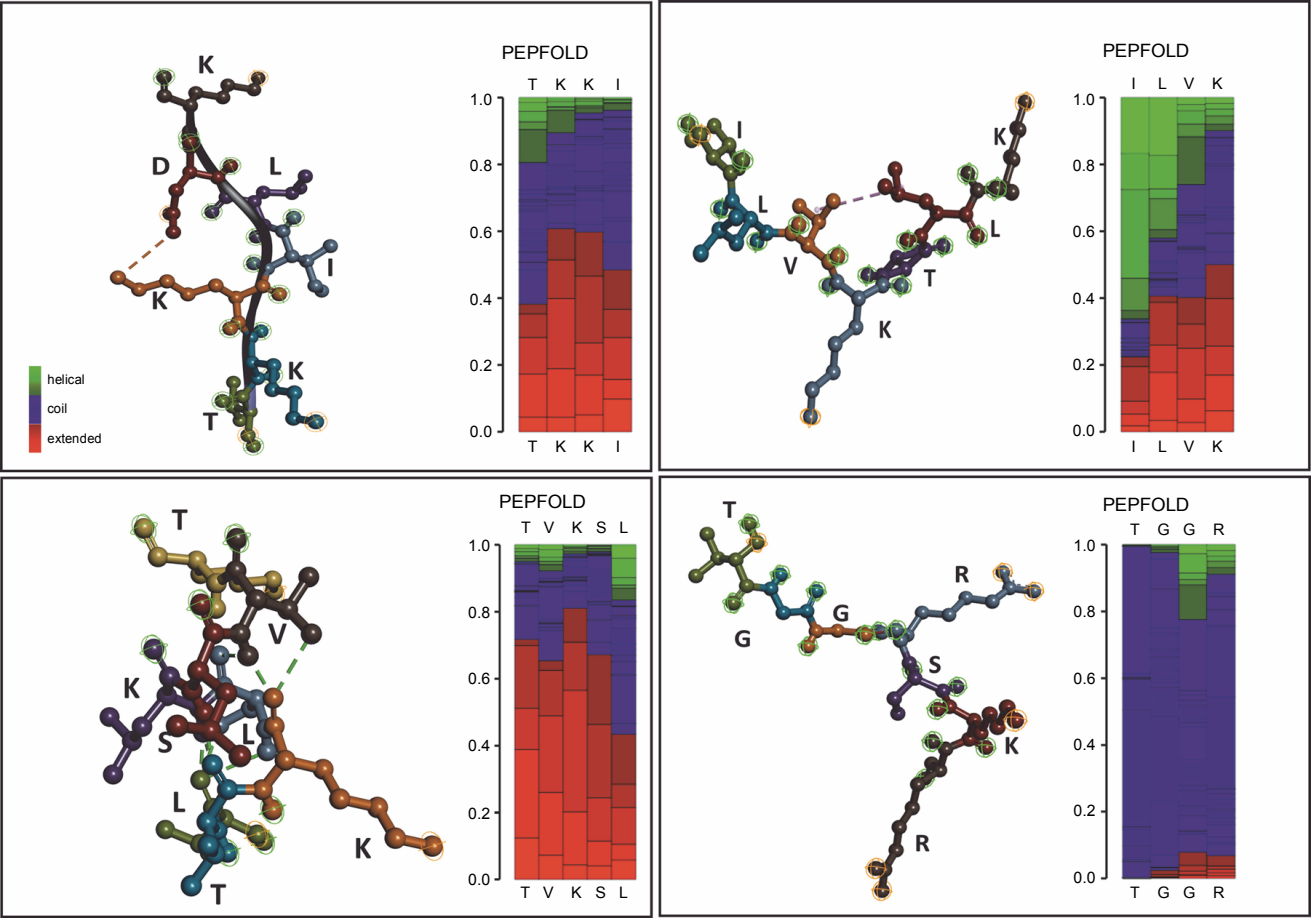
Amino acid sequence prediction models by using PEP-FOLD v4

In addition to unleashing *Gracilaria* spp. potency, a secondary structure model of amino acid sequence prediction was presented. We generated the model prediction to analyze the interactive site and particularly its basic conformation shown in Figure 5. Our secondary structure prediction is based on the Hidden Markov Model (HMM) by PEP-FOLD v4 (Camproux et al., 2004; Cegłowska et al., 2020; Nakamura-Gouvea et al., 2022; Temme et al., 2012). This model was used to discretize protein backbone conformation as a series of over-lapping fragments (states) of 4-residue length.

By using HMM, the model converts description of 3D structures from the geometric aspect towards a more explanatory description including local dependence information. This approach results in a both library of representative structural fragments and their first-order dependency, called Structural Alphabet (SA) (de Breven, et al. 2001; Camproux, Gautier and Tufféry, 2004). In this study, we found a high potency of hydrophilic interactions. Peptides with a β-sheet secondary structure have a surrounding hydrophilic group, making the peptide capable of targeting protein synthesis so that hydrogen bonds with the receptor are formed quite high. The coil or random coil model is formed from random or irregular hydrogen bonds. The formation of loops in the secondary structure of the α-helix and β-sheet is caused by the polar amino acid residues alanine, glycine, serine, and tyrosine. The formation of the loop results in a decrease in hydrogen bonds, so it is hydrophobic. Glycine is a polar amino acid and tends to form permanent loop patterns. Meanwhile, proline has the opposite property, namely hydrophilic (Salwiczek et al., 2012). Antimicrobial peptides often contain this looping structure, revealing its integration toward bacterial cell walls (Huan et al., 2020). This peptide conformation can then be proposed for drug development targets.

GP1 model confirmed its hydrophobic surface with the −H interactive site, generating spontaneous interactions with other peptides. Those properties justify its purpose as transporter peptides rRNA methyltransferase 2 mitochondrial RNA (Deng et al., 2022). However, the particular binding site for the methyl donor substrate remains unknown. In the GP2 (TVKSLLTK) case, the model showed conventional hydrogen interaction between amino acid active sites. GP3 supported the main characteristics of integral protein located in chloroplast, with the protein folding ability which supports strong conformation generated by hydrogen interactions. Therefore, the shape formed into coiled shapes. Similar to that, GP3 (ILVKTLK) showed one internal interaction for V and L sites, generating one coiled shape. Meanwhile, the coil shape of the secondary structure peptide was found in GP4 (TGGcRSKR) regardless of its modification site of carbamidomethyl. GP4 shaped unfold with high -H containing sites which releases hydrophobic amino acid segments. These polypeptides are highly prone to aggregation and the formation of unwanted interactions with other proteins (Ries et al., 2020). Since the GP4 is categorized as an uncharacterized peptide, we suggest that this conformation may lead to nascent polypeptides with high interactivity.

This active site analysis will open the possibility for further functional properties research. For instance, the adsorption of proteins to different surfaces, cell membranes, and implants in biological systems or heating during industrial processing, is often accompanied by proteins’ structural and conformational alteration (Lautenbach et al., 2021).
